# College of Physicians and Surgeons of the Iowa University, Session of 1855–6

**Published:** 1856-04

**Authors:** 


**COLLEGE OF PHYSICIANS AND SURGEONS OF THE IOWA UNIVERSITY, SESSION OF 1855-56.**

The commencement exercises of this Institution were held in the Atheneum of this city, on the evening of the 26th of February.

The Degree of Doctor of Medicine was conferred upon twenty-three by Prof. Allen, Dean, and the Valedictory address by Prof. M'Gugin, President of the Faculty.

The session was characterised throughout by harmony, and laborious application on the part of the class. Each one, with but one or two exceptions, seemed moved by an anxious desire to acquire, and emulous to excel. This was the only evidence of strife manifesting itself, for not an unkind feeling or personal difference occurred during the entire session. This made it profitable to the student, while it was most gratifying to the Faculty.

Each student most unequivocally exhibited the liveliest enthusiasm for the Institution, and a devotion to its best interests.

At the conclusion of the valedictory address, Dr. White, of the graduating class, resident of Missouri, replied extemporaneously in an impressive speech, in which he said that he represented the feeling and sentiment of the class, when he said that their devotion to school, and its present Faculty, would never abate and that the graduates, indeed the entire class, would ever remember with gratitude, the efforts made by their teachers, to advance them in acquirement, and the courteous attentions paid them during their past connexion with the College.

The following are the names of the graduates, their places of residence, and the subject of the Thesis of each:

| NAME. | RESIDENCE. | SUBJECT OF THESIS. |
|---|---|---|
| V. V. ADAMSON, | Iowa, | Miasmus, [Diseases. |
| J. BEERBOWER, | Iowa, | Remote Causes of Nervous |
| J. J. BROWN, | Mo., | Cerebral Apoplexy. |
| H. CARPENTER, | Iowa, | Circulation of the Blood. |
| J. W. DAVIDSON, | Iowa, | Cholera Infantum. |
| A. J. EVANS, | Iowa, | Malignant Cholera. |
| J. HENERY, | Mo., | Uterine Hemorrhage. |
| W. R. KINKADE, | Va. | Puerperal Convulsions. |
| J. W. LA FORCE, | Iowa, | Duties of Med. Attendance in |
| J. A. LADD, | N. Y., | Mania. [Natural Labor. |
| ROBT. MOORE, | Iowa, | Changes in the Blood. |
| D. R. MARTIN, | Iowa, | Placenta Previa. |
| F. B. REED, | Iowa, | Physiology of Digestion. |
| L. J. RHEA, | Ills., | Glandular Secretion. |
| J. M. SAYRES, | Ky., | Hygienic M'n'gem't of Ch'd'n. |
| F. M. STRATTON, | Ky., | Dysentery. |
| C. B STILLMAN, | Ills., | Effusion of Serum. |

to here

| NAME. | RESIDENCE. | SUBJECT OF THESIS. |
|---|---|---|
| J. K. TRIMBLE, | Iowa, | Gonorrhœa. |
| L. TURNER, | Mo., | Typhoid Fever. |
| J. S. VAN NEST, | Mo., | Etiology. |
| J. A. H. WHITE, | Mo., | Typhoid Fever. |
| F. M. WARFORD, | Iowa, | Calorification. |
| T. J. WILLIAMS, | Ohio, | Tetanus. |

Whole number of Matriculants, 79. Number of Graduates, 23.

The announcements for the coming session will duly and timely appear. The large size of the class of last session, the interest taken in the institution by the profession of the State, of contiguous territory of surrounding States, the presence in the class, of students as far east as New York, Pennsylvania, Virginia, and Ohio, together with the warm interest displayed by the citizens of this city, are guarantees of future prosperity and permanency.

